# PIEZO1 Is a Contextual Mechanosensor and Its Depletion in Scx‐Lineage Cells Accelerates Structural and Functional Recovery

**DOI:** 10.1096/fj.202602931R

**Published:** 2026-10-07

**Authors:** Nicole A. Chittim, Maja Wolleb, Maya Ben‐Yehuda Greenwald, Maja Bollhalder, Jess G. Snedeker

**Affiliations:** ^1^ Department of Orthopedics Balgrist University Hospital Zurich, University of Zurich Zurich Switzerland; ^2^ Institute for Biomechanics ETH Zurich Zurich Switzerland

**Keywords:** healing, mechanotransduction, PIEZO1, Scleraxis, tendon

## Abstract

Previous work has identified PIEZO1 as an important mechanosensor in homeostatic tendon, where it detects mechanical overload and regulates tissue adaptation. Here, we investigated the consequences of PIEZO1 depletion during tendon healing using a full‐thickness central‐third patellar tendon defect model. Unexpectedly, mechanical testing revealed accelerated functional recovery in mice with ScxCre‐driven *Piezo1* conditional knockout. Transmission electron microscopy and histological analyses further demonstrated accelerated structural recovery in ScxCre;Piezo1^f/f^ tendons. Bulk tissue transcriptomic analysis revealed overlapping core healing programs between mutant and wild‐type defect tendons, despite broader transcriptional remodeling in ScxCre;Piezo1^f/f^ tendons. However, ScxCre;Piezo1^f/f^ tendons showed a marked reduction in Scx^+^ cells within the defect region, consistent with prior reports suggesting that this subpopulation can adversely affect tendon healing. In vitro assays indicated that the reduced accumulation of Scx^+^ fibroblasts after *Piezo1* conditional knockout may be attributed to impaired migration rather than reduced proliferation. Together, these findings reveal a context‐dependent role for PIEZO1 in tendon biology: while PIEZO1 supports mechanosensing and adaptation in mature tendon, it enables wound‐site recruitment of Scx^+^ fibroblast populations that impair healing after injury.

## Graphical abstract

PIEZO1 supports adaptive matrix remodeling during tendon homeostasis but has a distinct role following injury. ScxCre‐driven *Piezo1* depletion reduced Scx^+^ cell recruitment/accumulation within the healing defect of the patellar tendon and was associated with accelerated collagen remodeling and improved mechanical recovery. These findings suggest that PIEZO1‐mediated mechanosensing may regulate recruitment of Scx‐lineage cells during tendon repair and demonstrate context‐dependent functions of PIEZO1 in tendon homeostasis and healing.

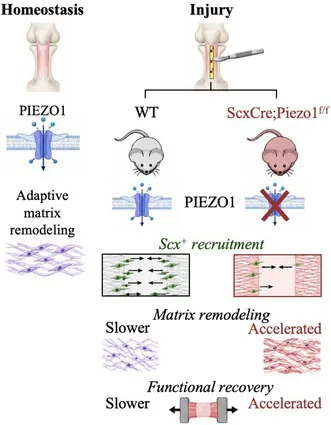

## 1 Introduction

Tendons are dense connective tissues that connect muscles to bones to facilitate movement [1, 2]. Tendon injuries account for approximately 50% of musculoskeletal injuries in the United States [3] and 30% of musculoskeletal injuries worldwide, with four million new cases each year [4]. Often, these injuries result from improper loading and, depending on their severity, may require surgical intervention for reattachment [3]. Regardless of surgical intervention, recovery from a tendon injury is slow and often incomplete, especially with regard to biomechanical recovery [3, 5].

Tendons are highly mechanosensitive tissues whose development, maintenance, and regeneration are regulated by mechanical loading [6]. Physiological tensile loading promotes collagen synthesis, matrix alignment, and tissue adaptation, whereas abnormal loading contributes to degeneration and failed repair [6]. Despite the central importance of mechanical cues in tendon biology, the molecular mechanisms by which tendon cells convert mechanical forces into reparative or maladaptive biological programs remain incompletely defined. Mechanical loading regulates multiple aspects of tendon healing, including inflammation, extracellular matrix synthesis and remodeling, collagen organization, angiogenesis, and stem/progenitor cell behavior [7, 8, 9]. Appropriate mechanical stimulation promotes collagen organization and functional recovery, whereas both stress deprivation and excessive loading impair tendon repair [7, 8, 9, 10]. Together, these observations indicate that successful tendon healing depends on the ability of resident tendon cells to appropriately sense and respond to mechanical cues [7].

Multiple mechanosensitive pathways have been implicated in regulating tendon development, homeostasis, and healing, including TGF‐β, Hippo pathway effectors YAP/TAZ, integrins, primary cilia, TRPV4 channels, and PIEZO ion channels [11, 12, 13, 14, 15]. Integrins mediate force transmission between the extracellular matrix and the cytoskeleton [12], whereas YAP/TAZ integrate changes in cell shape, substrate stiffness, and cytoskeletal tension into transcriptional responses [12, 13]. TRPV4 has been proposed to contribute to tendon mechanotransduction and matrix regulation [12, 14, 15], while primary cilia have emerged as important mechanosensory organelles in musculoskeletal tissues and are thought to contribute to tendon mechanobiology [12, 14]. However, these pathways are highly interconnected and share downstream intermediates, making it difficult to assign specific mechanical functions to individual sensors, particularly in the complex environment of tendon healing.

Among these mechanosensors, PIEZO1 is a stretch‐activated calcium ion channel that opens in response to mechanical stimuli through deformation of its trimeric, propeller‐like structure [16, 17]. We previously showed that PIEZO1 plays a central role in tendon mechanotransduction, functioning as a shear stress sensor and regulator of tendon extracellular matrix remodeling [17, 18]. In homeostatic tendon, PIEZO1 activity positively correlates with tendon stiffness and strength, and pharmacological PIEZO1 activation in tendon explants partially recapitulates the structural and functional effects of exercise [17]. Collectively, these findings identify PIEZO1 as an important mediator of load‐induced tendon adaptation, but whether PIEZO1 functions independently of, upstream of, or in concert with these other mechanosensory pathways during tendon repair is currently unknown.

Whether the same mechanotransductive programs that support tendon homeostasis also regulate tendon healing remains unresolved. This question has broad clinical relevance because tendon injuries are common and frequently heal poorly in mechanically dynamic environments [19], whether following acute trauma, overuse, or surgical intervention. To investigate these mechanisms, we employed a well‐established murine central‐third patellar tendon defect model, which recapitulates the tendon injury created during bone‐patellar tendon‐bone autograft harvest for anterior cruciate ligament (ACL) reconstruction [20, 21, 22, 23]. Clinically, healing of this defect remains imperfect and can contribute to donor‐site morbidity, including anterior knee pain, kneeling pain, extensor mechanism weakness, altered tendon remodeling, and, in rare cases, patellar fracture or tendon rupture. Understanding the molecular mechanisms that regulate tendon repair may therefore improve healing following tendon injury and surgical reconstruction.

In the present study, we aimed to explore the role of PIEZO1 in tendon healing using a full‐thickness central‐third patellar tendon injury model [24, 25, 26, 27] in *Piezo1*‐floxed mice crossed with a ScxCre driver (ScxCre;Piezo1^f/f^). Based on prior evidence that PIEZO1 activation supports tendon adaptation during homeostasis, we hypothesized that *Piezo1* depletion in Scx‐lineage tendon cells would impair healing. Surprisingly, *Piezo1* depletion accelerated both functional and structural recovery of mouse patellar tendons after 14 and 28 days of healing. Whole transcriptomic analysis revealed overlapping core healing programs, but broader transcriptional remodeling and stronger matrix‐organization signatures in ScxCre;Piezo1^f/f^ tendons. Unexpectedly, ScxCre;Piezo1^f/f^ tendons showed reduced accumulation of Scx^+^ cells within the defect region. Based on this finding, and on the known role of mechanosensing in cell migration and durotaxis, we tested whether PIEZO1 depletion impaired the migratory capacity of Scx‐lineage tendon cells. These findings support a model in which PIEZO has context‐dependent functions in tendon biology: while PIEZO1 promotes adaptive matrix remodeling in homeostatic tendon, it may also facilitate migration or recruitment of Scx^+^ cells that can delay structural and functional recovery after injury.

## 2 Materials and Methods

### 2.1 Mouse Line Generation

All mouse breeding and experiments were performed under the license ZH018/2022 (Switzerland) and carried out in accordance with Swiss and European animal welfare laws (EU Directive 2010/63/EU). We used Piezo1^f/f^ mice with a Scleraxis (Scx)‐Cre driver to produce a conditional knockout (KO) of *Piezo1* in Scx‐expressing cells. Validation of *Piezo1* conditional KO by immunostaining and RT‐qPCR is shown in Figure S1. We further intercrossed these mice with a ScxGFP reporter. Mice were housed in temperature and humidity‐controlled conditions, with a 12‐h light/dark cycle, with food and water available *ad libitum*.

### 2.2 In Vivo Patellar Tendon Injury Model Surgery

Only female mice were used for in vivo patellar defect experiments due to the improper healing observed in 80% of males. Mice aged 16–22 weeks old were weighed and assessed as healthy prior to surgery. Animals were treated with analgesia and anesthetized for the duration of the procedure. A vertical incision was made over the knee, and the patellar tendon was exposed and isolated. The central third of the patellar tendon was removed, the area was flushed with 0.9% NaCl, and the skin wound was sutured closed [24, 25, 26, 27]. The other leg was used as a contralateral sham: a skin incision was made, the patellar tendon was exposed and isolated, the area was flushed with 0.9% NaCl, and the skin wound was sutured closed. Mice were monitored and treated with analgesia post‐surgery. At specified endpoints, mice were euthanized via inhalation overdose with carbon dioxide (timeline in Figure 1A, schematic of surgery in Figure 1B).

**FIGURE 1:**
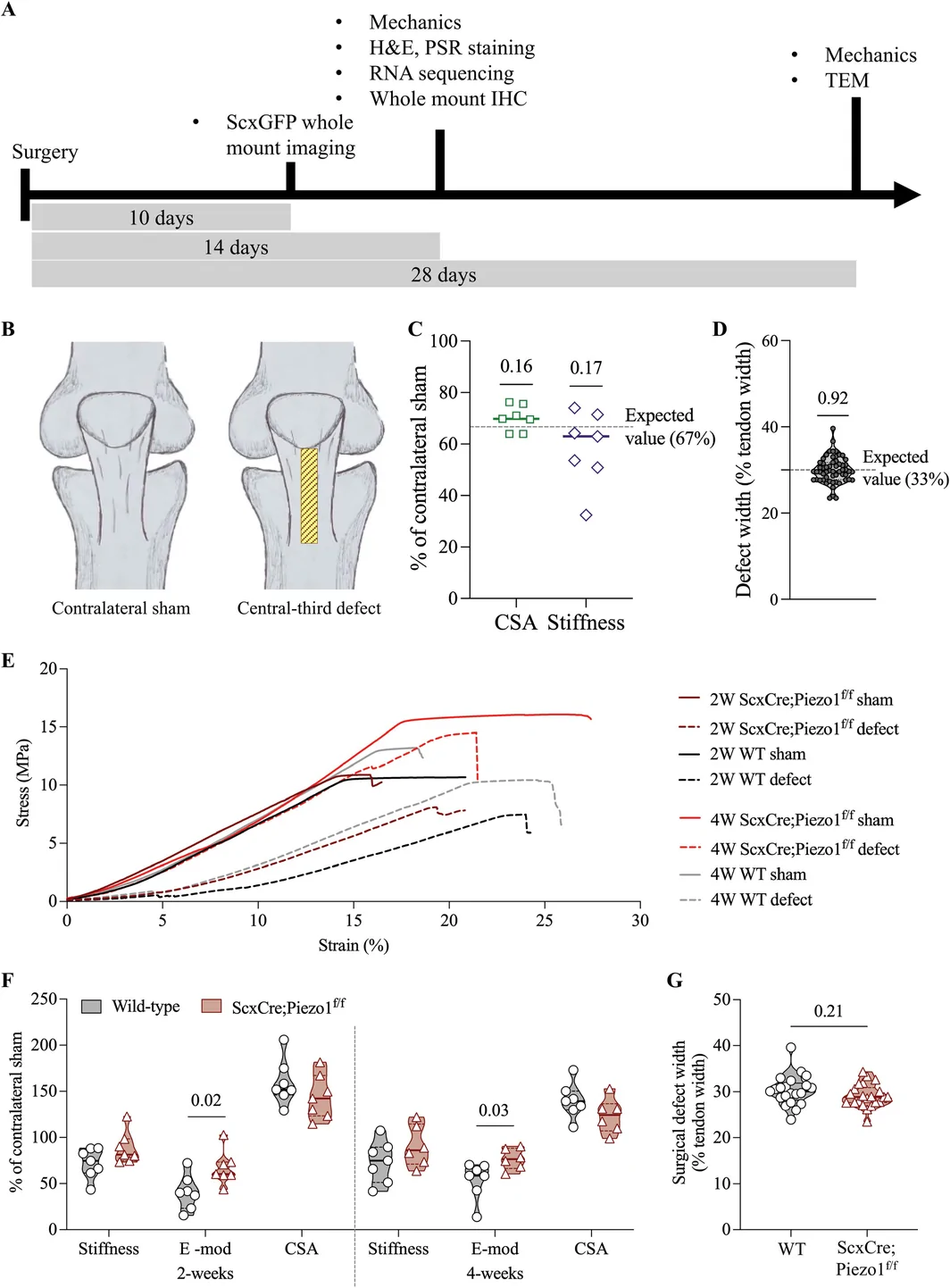
ScxCre;Piezo1^f/f^ mouse recovers functional properties faster than WT littermate controls. (A) Timeline of endpoint analyses for experiments. (B) Schematic of full‐thickness, central‐third patellar tendon injury model (excised region in yellow) while the contralateral leg is used as an intra‐animal sham control. (C) Mechanical analysis of the patellar tendon immediately following injury, reported as a percentage of the contralateral sham tendon. Each point represents one animal, *n* = 7. Statistics were performed using a one‐sample *t*‐test compared to a value of 67%, and *p*‐values are displayed. (D) Quantification of the defect width as a percentage of total tendon width for multiple surgeries. Each point represents one surgery/animal, *n* = 57 mice. Statistics were performed using a one‐sample *t*‐test compared to a value of 30%, and *p*‐value is displayed. (E) Representative stress–strain curves from sham and defect tendons from ScxCre;Piezo1^f/f^ and WT mice after both 2 and 4 weeks of healing. (F) Quantification of mechanical properties of defect tendons as a percentage of the contralateral sham tendon (*n* = 7 mice per group). (G) Quantification of surgical defect width of all mice as a percentage of total tendon width (*n* = 21 WT and 22 ScxCre;Piezo1^f/f^ mice). Statistics for mechanical analysis were performed with an unpaired Student's *t*‐test and *p*‐values are shown.

Endpoints were selected based on the biological questions addressed in each experiment and on previously established murine patellar tendon healing models. Mechanical testing and histological analyses were performed after 2 and 4 weeks of healing because these timepoints correspond to early and intermediate stages of tendon repair and are widely used for assessing structural and functional recovery [24, 25]. RNA sequencing and immunohistochemical analyses were performed after 2 weeks to capture active tissue remodeling before substantial matrix maturation. ScxGFP imaging was performed 10 days after injury because previous studies have identified this period as corresponding to peak migration and accumulation of *Scx*‐expressing cells within the healing defect [28].

### 2.3 Patellar Tendon Isolation and Mechanical Testing

For mechanical analysis, 7 mice per genotype per timepoint were analyzed. Mice were euthanized at either 2 or 4 weeks post‐surgery, and the patellar tendon (PT) unit was excised as patella bone—patellar tendon—tibia. The width and depth of the tendon were measured prior to testing using an inverted microscope (Motic AE2000, 4× objective) and calibrated imaging software (Motic Images Plus 3.0). The cross‐sectional area was calculated assuming a rectangular cross‐section. Mechanical testing was performed using the custom fixture and loading configuration previously described by Chittim et al. [29]. Briefly, the patella‐patellar tendon‐tibia unit was mounted by securing the patella and tibia in custom grips fitted to a universal testing machine, Zwick, allowing tensile loading to be applied along the longitudinal axis of the tendon while maintaining the native tendon‐bone insertions. The unit was submerged in 1% PBS for the duration of the test: preloading to 0.5 N, preconditioning to 1% strain for 25 cycles, resting for 30 s, and finally stretching to failure at a rate of 5.0% L_0_/s [30]. The machine was fitted with a 20 N load cell, and force and position data were recorded. Force data and tendon cross‐sectional area were used to calculate stress, while position data were used to calculate strain. Elastic modulus was calculated as the slope of the linear region of the stress–strain curves, the failure stress as the maximum stress, and the failure strain as the corresponding strain value [31].

### 2.4 TEM Preparation and Imaging

Four weeks post‐surgery, a subgroup of mice was euthanized (3 mice per genotype), and patellar tendons were isolated. Fixation buffer was prepared fresh: 50% 0.2 M sodium cacodylate buffer and 2.5% glutaraldehyde in H_2_O. Tendons were fixed in fixation buffer at room temperature for 1 h, and stored at 4°C for at least 24 h. Samples were transferred to the Center for Microscopy and Image Analysis at the University of Zurich, where they were washed in cacodylate buffer three times. Samples were post‐fixed in 1% osmium tetroxide for 40 min at RT, stained with 1% uranyl acetate for 1 h at RT in the dark, and dehydrated through a graded ethanol series for 20 min each. Samples were then embedded in epoxy resin (66% Epon/Araldite for 1 h, 100% Epon/Araldite for 1 h, polymerization at 60°C overnight), sectioned into ultrathin sections of 70 nm, and contrast‐enhanced with lead citrate for 5 min prior to imaging under transmission electron microscopy (TEM). Resulting images were provided with anonymous sample identifiers, and quantitative analysis was performed by a single investigator blinded to the genotype throughout the analysis. Fibril segmentation was performed in FIJI (2.16.0/1.54p) using the StarDist plugin. The resulting regions of interest were used to quantify fibril diameter distributions and fibril density. More than 1000 collagen fibril cross‐sectional areas were quantified from more than 10 image fields per group across three separate mice. Collagen area density was calculated as the area covered in collagen fibrils as a percentage of the total area analyzed. More than 10 image fields were analyzed and averaged for each group across three separate mice.

### 2.5 Embedding, Sectioning, and Histology

After 2 weeks of healing, mice were euthanized (3 mice per genotype for each stain), and hindlimbs were fixed intact in a standardized 90° knee‐joint configuration in 4% formalin for 24 h. Following fixation, limbs were washed, dissected further, and transferred to the Musculoskeletal Research Unit (MSRU), Department of Molecular Mechanisms of Disease, University of Zurich (Zurich, Switzerland) for decalcification in 14% EDTA at 4°C for approximately 2 weeks, until the bone was sufficiently decalcified. Samples were subsequently paraffin embedded and sectioned. Serial sections were stained with hematoxylin and eosin (H&E) and Picrosirius red (PSR) according to the manufacturers' protocols. H&E stained sections were analyzed in FIJI 2.16.0/1.54p by a single investigator blinded to genotype using coded images. Thresholding was used to define total tendon tissue area, and cell nuclei were subsequently identified using color thresholding. The number of cells was quantified using FIJI's Analyze Particles function, and cellularity was calculated as the number of cells present per total tendon tissue area. PSR stained sections were imaged under polarized light microscopy and analyzed in FIJI 2.16.0/1.54p. Individual color area was quantified as a percentage of total tendon tissue area using the standardized hue thresholds described by Rich and Whittaker [32], which classify birefringence into red (2–9, 230–256), orange (10–38), yellow (39–51), and green (52–128) hue ranges.

Additional fixed, dissected, and decalcified hindlimbs were embedded in OCT and cryosectioned (7 μm sections). These sections were used for immunohistochemistry of vimentin, Piezo1, and Ki67. A list of antibodies and buffers is available in Supporting Information. Briefly, the sections were thawed and dried, fixed with 4% PFA for 10 min and then washed with PBS. Sections were blocked with 3% BSA in PBST for 1 h and incubated overnight at 4°C with primary antibodies. Samples were washed, incubated for 1 h with secondary antibodies, and washed again in PBS. Samples were incubated for 20 min with NucBlue and washed again in PBS before being quickly washed with deionized water and having coverslips mounted. Imaging was performed using a Nikon Eclipse Ti2 inverted microscope (60× with lasers at 405 nm, 546 nm, and 647 nm).

### 2.6 RNA Extraction and Sequencing

Mice were euthanized 2 weeks post‐surgery, and patellar tendons were immediately extracted and snap‐frozen in liquid N_2_. Because the RNA yield from a single murine patellar tendon was insufficient for library preparation, patellar tendons from two mice within the same experimental group were pooled to generate one biological sample (12 mice per genotype for a total of 6 samples per genotype and condition). Injured tendons were pooled separately from the corresponding sham tendons. The frozen tissue was milled and homogenized with Genezol. The tissue lysate was incubated on ice for 10 min with chloroform and then centrifuged at 4°C for 15 min. The supernatant was then used with the RNeasy Micro Kit (Qiagen, 74004) following the manufacturer's instructions. RNA concentration was measured using a Biotek microplate reader, and samples were sent to GENEWIZ (Leipzig, Germany) for RNA sequencing. RNA‐seq libraries were prepared using total RNA with PolyA selection enrichment and then sequenced with a NovaSeq 6000 sequencer (Illumina Inc., USA) using a sequencing depth of ~20 million paired‐end reads per sample.

### 2.7 Bioinformatics Analysis

Primary‐level bioinformatics analysis was performed on the SUSHI platform [33]. FastQC was used to verify the quality of the raw RNAseq data [34], and the raw.fastq files were then loaded to the STAR aligner software [35] to map the data to the *Mus musculus* genome reference 01‐30 (GENCODE GRCm39—Release_M31‐2023‐01‐30) and to calculate read counts per sample with the featureCounts function. Differentially expressed genes were computed using the DESeq2 package in the SUSHI environment with contrasts defined as defect vs. sham control (R version Dev/R/4.5.0). Mapped read counts were then analyzed using the EdgeR package for differential expression analysis [36], which was further supplemented by pathway analysis (cutoff FDR < 0.05, log_2_FC > 1 for all DEGs) using EnrichR [37]. Computational prediction of cell type fractions was performed using the CIBERSORTx digital convolution tool [38] and cell‐type signature matrix of de Micheli et al. [39]. Because a comparable single‐cell reference dataset for the murine patellar tendon was not available at the time of analysis, the Achilles tendon dataset was used as the reference for cellular deconvolution.

### 2.8 Whole Mount Immunostaining Protocol

Mice were euthanized 2 weeks post‐surgery (6 mice per genotype), and each patellar tendon was isolated and embedded in a small amount of 2% agarose. Once the agarose had hardened, the tendon was fixed in 4% formalin for 1 h, and then a previously defined protocol for whole mount immunostaining was followed [40]. Briefly, antigen retrieval was performed for 6 h at 4°C. Samples were blocked in blocking buffer for 20 min at 37°C. Samples were incubated overnight at 37°C with the appropriate primary antibody solution (Table 1), followed by washing for 1 h at 37°C, with the wash buffer changed every 15 min. Samples were incubated for 4 h at 37°C with the corresponding secondary antibody solution (Table 1). Samples were incubated with NucBlue in PBS for 20 min at 37°C and then washed with wash buffer for 1 h at 37°C before being dehydrated in a gradient of isopropanol (30%, 50%, 80%) for 10 min each and 100% for 30 min at room temperature. Samples were cleared with ethyl cinnamate for 20 min at room temperature and mounted on a glass coverslip for imaging, which was performed using a Nikon Eclipse Ti2 inverted microscope (lasers at 405 nm, 488 nm, 546 nm, and 647 nm). Primary and secondary antibodies used for each staining experiment are listed in Table 1, and the compositions of the buffers used are provided in the Supporting Information.

**TABLE 1:** Antibodies used for immunohistochemistry.

Primary Ab	Manufacturer	Concentration	Secondary Ab	Manufacturer	Concentration
Human/Mouse/Rat CD31/PECAM‐1 Antibody	R&D Systems, AF3628	10 μg/mL	Donkey anti‐goat IgG (H + L) Cross‐Adsorbed Secondary Antibody, Alexa Fluor 647	ThermoFisher Scientific, A21447	1:250
Anti‐CD68 Recombinant Multiclonal antibody	Abcam, RM1031	1:75	Donkey anti‐rabbit IgG (H + L) Cross‐Adsorbed Secondary Antibody, Alexa Fluor 488	ThermoFisher Scientific, A21206	1:250
CD206 Monoclonal Antibody (MR5D3)	ThermoFisher Scientific, MA5‐16868	1:100	Goat anti‐rat IgG (H + L) Cross‐Adsorbed Secondary Antibody, Alexa Fluor 546	ThermoFisher Scientific, A21247	1:250
Vimentin Monoclonal Antibody (V9)	Santa Cruz Biotechnology, sc‐6260	1:200	Donkey anti‐mouse IgG (H + L) Cross‐Adsorbed Secondary Antibody, Alexa Fluor 546	ThermoFisher Scientific, A10036	1:250
Piezo1 Monoclonal Antibody	ThermoFisher Scientific, MA5‐32876	1:100	Donkey anti‐mouse IgG (H + L) Cross‐Adsorbed Secondary Antibody, Alexa Fluor 546	ThermoFisher Scientific, A10036	1:250
Anti‐Ki67 Antibody	Abcam 15 580	1:200	Goat anti‐rabbit IgG (H + L) Cross‐Adsorbed Secondary Antibody, Alexa Fluor 647	ThermoFisher Scientific, A21244	

### 2.9 Whole Mount ScxGFP Imaging

Mice were euthanized 10 days post‐surgery (6 mice per genotype), and each patellar tendon was isolated and fixed in 4% formalin for 1 h. Tendons were washed with 1% PBS and incubated for 20 min at room temperature with NucBlue. After incubation, they were washed again with 1% PBS and placed on a glass slide for imaging (Nikon Eclipse Ti2 inverted microscope, using lasers at 405 nm and 488 nm).

### 2.10 Cell Isolation, Transfection, and Culture

Mice were euthanized, and patellar tendons were isolated. Patellar tendons were incubated at 37°C rotating overnight with 2 mg/mL collagenase in Dulbecco's Modified Eagle Medium (DMEM) with 1% Penn/Strep. Once digested, the samples were centrifuged for 5 min at 0.5 rcf. Excess medium was aspirated, and cells were resuspended and plated with DMEM supplemented with 1% Penn/Strep and 10% fetal bovine serum.

### 2.11 In Vitro Scratch Assay

A scratch assay protocol was developed based on previous literature [41, 42, 43, 44]. Cells isolated from individual mice were maintained as independent biological replicates throughout the experiment, with *n* = 4 mice per experimental condition depending on the analysis. Three technical replicate wells were analyzed per mouse, and the values from these wells were averaged to obtain a single value for each biological replicate. Cells at p3–p5 were plated at 250 000 cells/mL in collagen‐coated wells of a 12‐well plate. Plates were incubated at 37°C until cells were confluent. Medium was removed and replaced with 1% FBS medium, and the cells were incubated for 24 h to arrest proliferation. A p200 pipette tip was used to scratch a continuous line through the center of each well. Each well was washed three times with PBS to remove any floating cells. Medium without phenol red and containing 1% FBS was added back to the wells, and each well was imaged directly at time 0. Wells were incubated at 37°C and imaged after 4, 8, 12, and 24 h. At 48 h, cells were incubated for 20 min with NucBlue and imaged.

### 2.12 Statistics

All statistical analyses were performed using GraphPad Prism (v. 10.6.1). Sample sizes were determined based on previous experience with this murine patellar tendon injury model in our laboratory and published studies using similar models. For the mechanical testing cohorts (*n* = 7 mice per genotype and timepoint), sample size was additionally informed by a priori power calculations designed to achieve 80% statistical power with α = 0.05. Prior to parametric analyses, normality was assessed graphically using Q–Q plots, and homogeneity of variance between groups was assessed using *F* tests in GraphPad Prism. No significant differences in variance were detected for datasets analyzed using unpaired Student's *t*‐tests. Mechanical testing data, TEM measurements, cellularity, and ScxGFP quantification met the assumptions for parametric analysis and were analyzed using unpaired Student's *t*‐tests. For mechanical testing, the value obtained from the injured tendon of each mouse was normalized to the corresponding contralateral sham tendon, yielding one defect‐to‐sham ratio per animal. WT and ScxCre;Piezo1^f/f^ mice were compared independently at each healing timepoint. CD31, CD68, CD206, and vimentin immunohistochemistry data did not meet the assumptions for parametric analysis and were therefore analyzed using nonparametric tests (Mann–Whitney). PSR birefringence data were analyzed using a two‐way ANOVA to compare the distribution of birefringence colors between genotypes. Statistical analysis of RNA sequencing data was performed using the adjusted *p*‐value from the differential expression through the Functional Genomics Center Zurich SUSHI framework (ETH Zurich and University of Zurich) [33]. In vitro scratch assay analysis was performed with Student's *t*‐tests with a correction for multiple comparisons when applicable.

## 3 Results

### 3.1 Loss of PIEZO1 Accelerates Functional Recovery of Patellar Tendons After Injury

Efficient *Piezo1* conditional KO in ScxCre;Piezo1^f/f^ tendons was confirmed by immunostaining and RT‐qPCR (Figure S1). We next verified that our surgical defect model and mechanical testing protocol produced a consistent and reproducible injury. Immediate post‐surgical testing demonstrated an approximate one‐third reduction in cross‐sectional area (CSA) and stiffness (Figure 1C, *p* = 0.16 and 0.17, respectively), consistent with excision of the central third of the tendon. The surgical defect width was also highly reproducible between animals, supporting the consistency of the injury model (Figure 1D, *p* = 0.92).

We performed ramp‐to‐failure tensile tests on sham and defect patellar tendons of WT and ScxCre;Piezo1^f/f^ mice after 2 and 4 weeks of healing (representative stress–strain curves Figure 1E). We found that, after both 2 and 4 weeks of healing, ScxCre;Piezo1^f/f^ mouse defect tendons had significantly higher elastic moduli when normalized to the contralateral sham than those from WT mice (*p* = 0.02 and 0.03, respectively, Figure 1F). Stiffness showed a similar trend toward higher values in ScxCre;Piezo1^f/f^ tendons at both timepoints but did not reach statistical significance. It is also important to note that there was no difference in surgical defect width (*p* = 0.21, Figure 1G). Normalized cross‐sectional area also did not differ significantly between genotypes at either timepoint.

Taken together, these analyses suggest that the ScxCre;Piezo1^f/f^ mice recovered mechanical competence of their tendons after surgical injury faster than the WT. Because this mechanical improvement occurred without genotype‐dependent differences in CSA, we next asked whether *Piezo1* depletion altered collagen organization within the healing defect.

### 3.2 Loss of PIEZO1 Accelerates Structural Recovery of Patellar Tendons After Injury

Because ScxCre;Piezo1^f/f^ tendons showed accelerated functional recovery without genotype‐dependent differences in CSA, we next asked whether *Piezo1* depletion altered collagen organization within the healing tendon. We performed transmission electron microscopy (TEM) imaging of tendon cross‐sections 4 weeks after surgery (representative images Figure 2A). Fibril diameter distributions showed similar bimodal profiles across groups, with a nonsignificant shift toward a greater frequency of larger fibrils in ScxCre;Piezo1^f/f^ tendons (Figure 2B). Although fibril diameter did not differ significantly between genotypes, collagen fibril density within the defect region was significantly higher in ScxCre;Piezo1^f/f^ tendons than in WT tendons (*p* = 0.04, Figure 2C), whereas fibril density in the intact tendon struts was similar between groups. These findings suggest that *Piezo1* depletion enhances collagen packing within the healing defect region. We therefore next investigated whether these structural changes were also consistent histologically.

**FIGURE 2:**
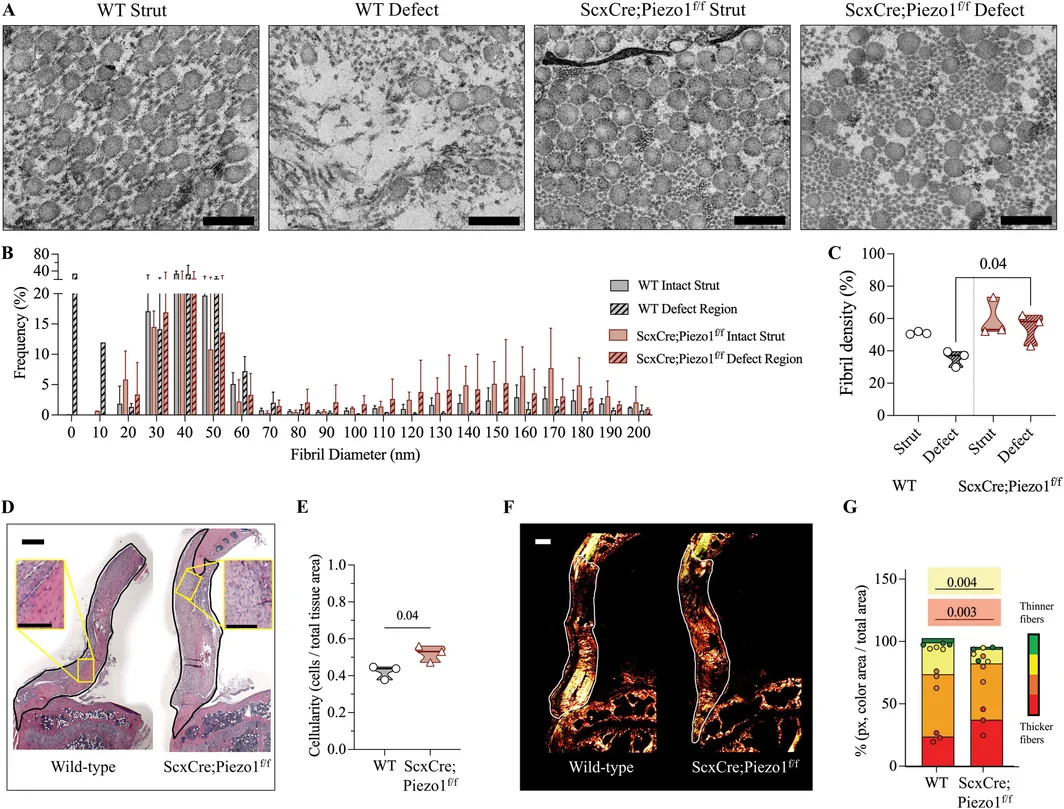
ScxCre;Piezo1^f/f^ mouse recovers structural properties faster than WT littermate controls at 2 and 4 weeks of healing. (A) Representative TEM images after 4 weeks of healing. Scale bar represents 500 nm. (B) Histogram of collagen fibril diameter. Bars represent mean ± SD (> 1000 fibrils each from > 10 areas from 3 mice per group). (C) Quantification of fibril density as a percentage of total area (*n* = 3 mice per group, each point represents an average of > 10 separate areas). (D) Representative images of H&E staining of the defected area of the tendons from ScxCre;Piezo1^f/f^ and WT mice after 2 weeks of healing with higher magnification pop out. Scale bar represents 400 μm. (E) Quantification of cellularity as the number of cells per total tissue area (*n* = 3 mice, each point represents an average of 3 sections). (F) Representative images of PSR staining under polarized light microscopy of the defected area of the tendons from ScxCre;Piezo1^f/f^ and WT mice after 2 weeks of healing. Scale bar represents 400 μm. (G) Quantification of individual color areas as a percentage of total tissue area (*n* = 3 mice, each point represents an average of three sections). Hue thresholds: Red 2–9, 230–256, orange 10–38, yellow 39–51, green 52–128. Statistics for panels (C) and (E) were performed with an unpaired Student's *t*‐test, for panel G with a 2‐way ANOVA, and *p*‐values are shown.

Patellar tendons were also isolated 2 weeks after surgery for histological analysis by H&E and Picrosirius red (PSR) staining. Representative images from the central healing defect region are shown in Figure 2D,F, respectively. Quantification of H&E‐stained sections within the defect region showed higher cellularity, defined as cell number normalized to total tendon tissue area, in ScxCre;Piezo1^f/f^ tendons compared with WT tendons (*p* = 0.04, Figure 2E). PSR‐stained sections from the defect region were then imaged under polarized light to assess collagen fiber organization. ScxCre;Piezo1^f/f^ tendons showed a significantly larger red birefringent area, consistent with increased abundance of thicker and/or more mature collagen fibers (*p* = 0.003, Figure 2G). Conversely, WT tendons showed a significantly larger yellow birefringent area, consistent with a greater abundance of thinner and/or less mature collagen fibers (*p* = 0.004, Figure 2G).

Together with the TEM findings, these histological analyses indicate accelerated structural remodeling in ScxCre;Piezo1^f/f^ tendons following injury.

### 3.3 *Piezo1* Depletion Preserves Core Healing Programs While Enhancing Collagen Organization Signatures

After discovering that the loss of PIEZO1 in mouse tendons accelerates both the functional and structural recovery of the tendon after injury, we investigated the biological processes behind this phenomenon. For analysis purposes, we examined the ScxCre;Piezo1^f/f^ defect tendons compared to corresponding sham tendons, and the WT defect tendons compared to corresponding sham tendons. When looking only at the WT mice, we found 229 upregulated DEGs and 156 downregulated DEGs in the defect tendon when compared to the contralateral sham control (Figure 3A). For the ScxCre;Piezo1^f/f^ mice, we found 978 upregulated DEGs and 1251 downregulated DEGs in the defect tendon compared to the sham (Figure 3B). We then focused on upregulated genes only in the defect tendons compared to the shams (cutoff FDR < 0.05, log_2_FC > 0) and used EnrichR to identify several gene ontology (GO) terms and hallmark pathways.

**FIGURE 3:**
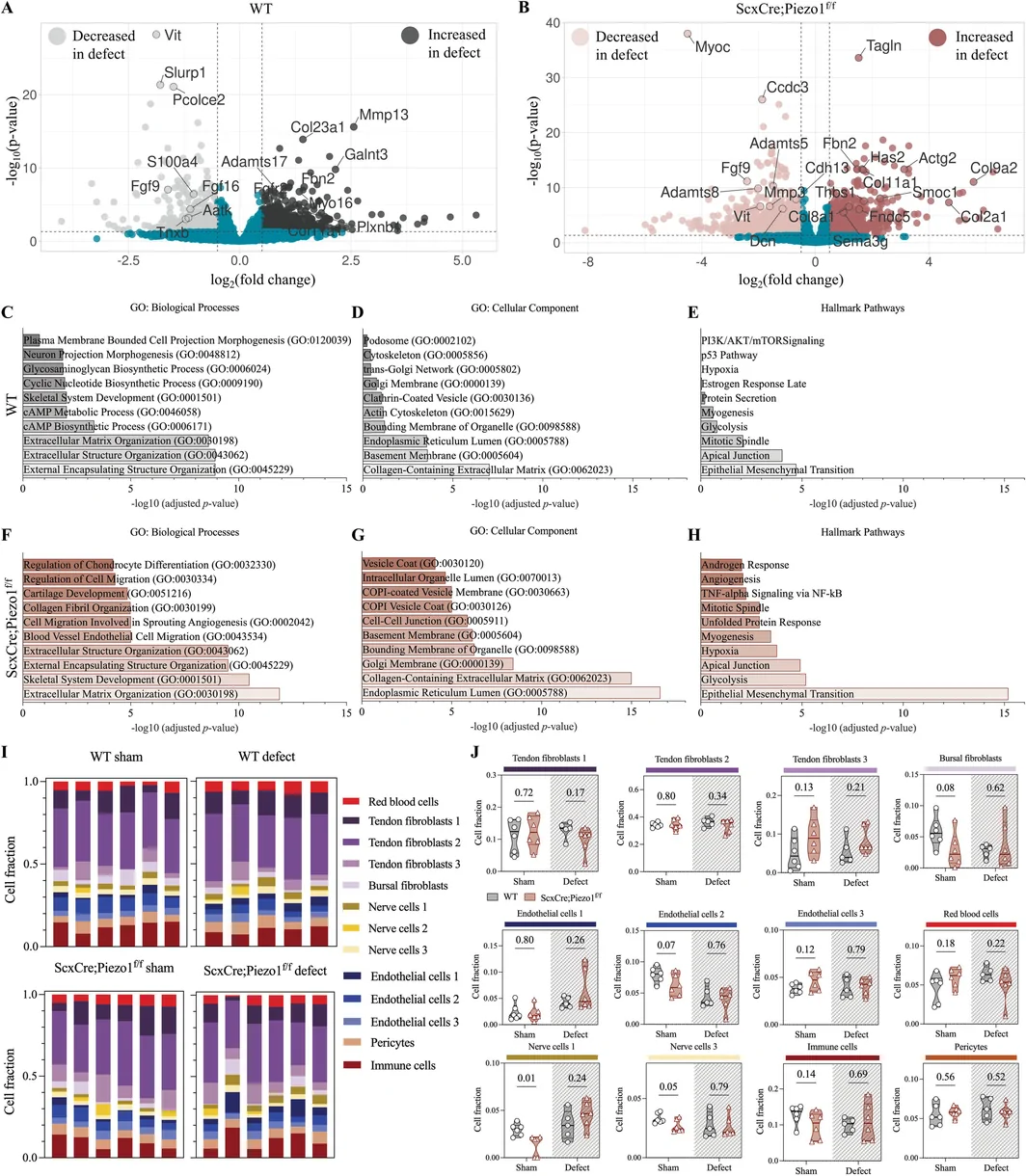
Whole transcriptomic analysis of defect tendons compared to contralateral sham control shows overlapping healing programs with stronger transcriptional remodeling in ScxCre;Piezo1^f/f^ tendons after 2 weeks of healing. (A) Volcano plot of RNA sequencing DEGs of WT and (B) ScxCre;Piezo1^f/f^ defect versus sham, identifying significantly expressed genes. (C) Top 10 predicted upregulated Biological Processes Gene Ontology terms for WT and (F) ScxCre;Piezo1^f/f^ mouse defect tendons compared to sham. (D) Top 10 predicted upregulated Cellular Component Gene Ontology terms for WT and (G) ScxCre;Piezo1^f/f^ mouse defect tendons compared to sham. (E) Top 10 predicted upregulated Hallmark Pathways for WT and (H) ScxCre;Piezo1^f/f^ mouse defect tendons compared to sham. (I) CIBERSORTx digital deconvolution analysis of cellular composition for individual sham and defect patellar tendons (each bar represents one sample). (J) CIBERSORTx digital deconvolution analysis of specific cell types. Each point represents one sample, *p*‐value is shown (*n* = 6 WT sham, 5 WT defect, 6 ScxCre;Piezo1^f/f^ sham, and 6 ScxCre;Piezo1^f/f^ defect).

Many biological processes GO terms were shared among the WT and ScxCre;Piezo1^f/f^ defect tendons when compared to corresponding sham controls, including terms such as extracellular matrix organization, extracellular structure organization, external encapsulating structure organization, and skeletal system development (Figure 3C,F, respectively). Biological processes identified as upregulated only in the WT defect tendons include cAMP biosynthesis and metabolic processes, glycosaminoglycan biosynthesis, and neuron projection. Those identified as upregulated only in the ScxCre;Piezo1^f/f^ defect tendons include endothelial cell migration and collagen fibril organization. Many collagen types were included in the upregulated DEGs in the ScxCre;Piezo1^f/f^ defect tendons compared to shams, some of which are highlighted in the volcano plot (Figure 3B). When examining cellular component GO terms, we again found many shared terms: collagen‐containing ECM, basement membrane, Golgi membrane, and endoplasmic reticulum lumen. Cellular components upregulated only in WT defect tendons included those related to the cytoskeleton (Figure 3D), whereas those in ScxCre;Piezo1^f/f^ defect tendons included those related to COPI (Figure 3G). Upregulated shared hallmark pathways between the two groups included glycolysis, mitotic spindle, and hypoxia. Upregulated pathways unique to the WT group included the PI3K/AKT/mTOR signaling pathway (Figure 3E). Those unique to the ScxCre;Piezo1^f/f^ group included TNF‐α signaling via NF‐kB (Figure 3H).

CIBERSORTx digital cytometry was also performed using a previously published single‐cell RNA sequencing dataset from murine Achilles tendon [39] as the reference dataset to estimate relative abundance of cell subpopulations within each bulk RNA sequencing sample (Figure 3I). The predicted cell populations correspond to transcriptionally distinct clusters defined in the reference single‐cell dataset [39]. The fibroblast populations were characterized by moderate‐to‐high *Col1a1* expression but differed in their expression of additional extracellular matrix‐associated genes, with tendon fibroblast 1 enriched for *Spp1*, tendon fibroblast 2 enriched for *Dpt*, and tendon fibroblast 3 corresponding to the *Col22a1*‐enriched junctional fibroblast population described in the reference dataset. When looking at individual cell subpopulations, we found that nerve cells 1 and 3 were significantly lower in ScxCre;Piezo1^f/f^ sham tendons than in WT sham tendons (Figure 3J, *p* = 0.01 and 0.05, respectively). Apart from reduced predicted nerve cell populations in ScxCre;Piezo1^f/f^ sham tendons, CIBERSORTx did not identify significant genotype‐dependent differences in predicted cell fractions within defect tendons.

Taken together, these data indicate that ScxCre;Piezo1^f/f^ and WT tendons activate overlapping core healing programs after injury, while *Piezo1*‐depleted tendons show broader transcriptional remodeling and selective enrichment of collagen fibril organization pathways.

### 3.4 Infiltration of Macrophages and Endothelial Cells Into Central‐Third Defect Is Similar in ScxCre;Piezo1^f/f^ and WT Mice

H&E histological data indicated a higher cell density in the defect region of the tendons from ScxCre;Piezo1^f/f^ mice, and the biological analyses predicted similar cell populations in the defect tendons from both the ScxCre;Piezo1^f/f^ and WT mice. Therefore, we sought to further explore specific cell types to investigate the potential causes of the healing phenotype we observed. To do this, we performed whole‐mount immunohistochemistry for CD31, CD68, and CD206, in addition to staining with NucBlue (representative images in Figure 4A,B). When we compared the normalized values from the WT mice to those from the ScxCre;Piezo1^f/f^ mice, we found no significant differences in the amounts of CD31^+^ cells (Figure 4C, *p* = 0.89), CD206^+^ cells (Figure 4D, *p* = 0.54), or CD68^+^ cells (Figure 4E, *p* = 0.61).

**FIGURE 4:**
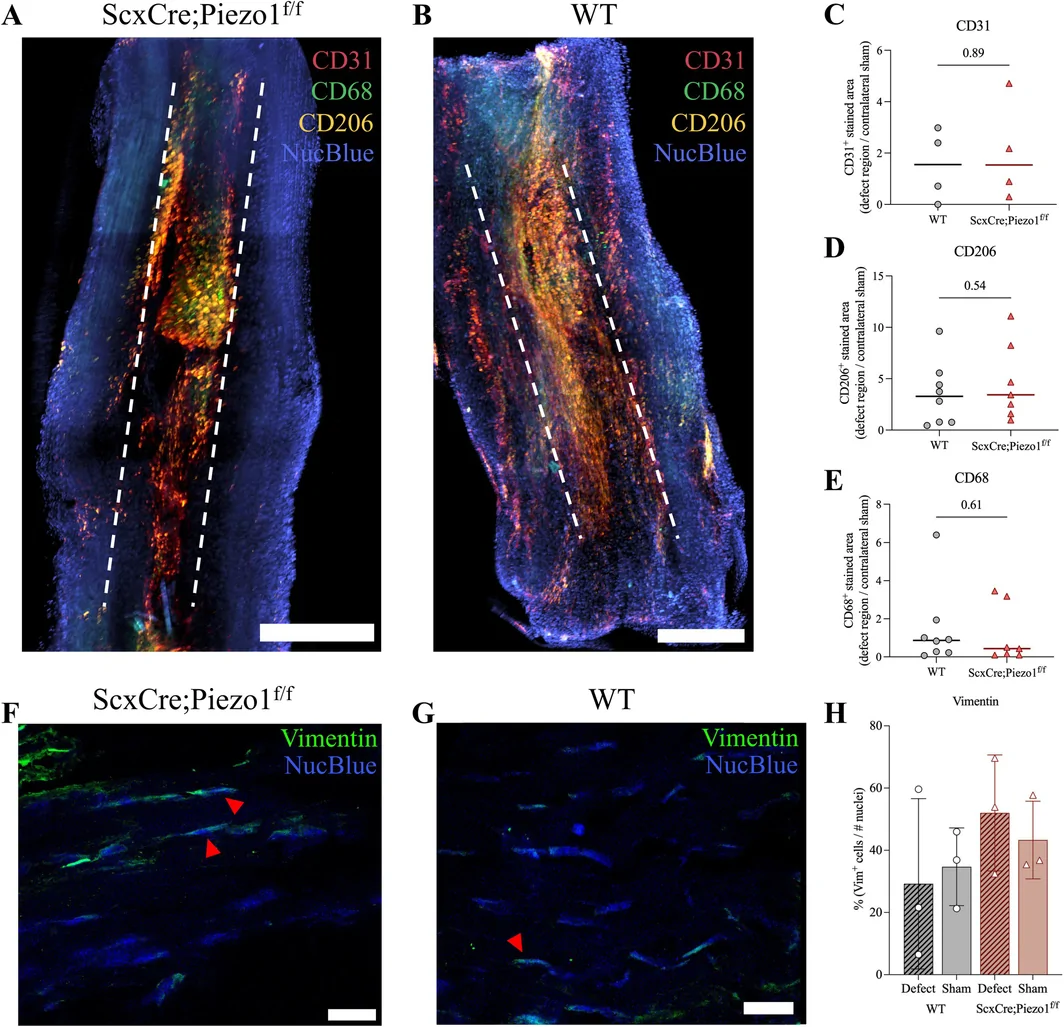
Whole mount immunostaining reveals similar amounts of endothelial cells and macrophages in defect region of tendons, whereas cryosection immunostaining shows increased numbers of vimentin^+^ cells in ScxCre;Piezo1^f/f^ defect tendons. (A) Representative z‐projection image of ScxCre;Piezo1^f/f^ defect tendon. (B) Representative z‐projection image of WT defect tendon. Scale bars represent 400 μm. Red is CD31, green is CD68, yellow is CD206, blue is NucBlue. Dashed white line indicates the surgical defect. (C) Quantification of CD31^+^ stained area in defect region normalized to corresponding sham control (*n* = 4). (D) Quantification of CD206^+^ stained area in defect region normalized to corresponding sham control (*n* = 8 WT and 7 ScxCre;Piezo1^f/f^). (E) Quantification of CD68^+^ stained area in defect region normalized to corresponding sham control (*n* = 8 WT and 7 ScxCre;Piezo1^f/f^). (F) Representative images of ScxCre;Piezo1^f/f^ defect tendon and (G) WT defect tendon. Scale bars represent 20 μm. Nuclei are represented in blue and vimentin^+^ nuclei are surrounded by green. Red triangles indicate vimentin^+^ cells. (H) Quantification of vimentin^+^ cells as a percentage of nuclei. Bars are represented as average ± standard deviation. Each point represents an average of four technical replicates for one mouse (*n* = 3).

These data indicate that the accelerated healing phenotype in ScxCre;Piezo1^f/f^ tendons was not associated with detectable differences in CD31^+^ endothelial area, CD68^+^ macrophage area, or CD206^+^ macrophage area within the defect region.

### 3.5 Cellular Infiltration Into Defect Region Exhibits Fibroblast Markers

H&E histological data indicated a higher cell density in the defect region of the tendons from ScxCre;Piezo1^f/f^ mice, but whole mount immunohistochemistry identified similar cell populations of endothelial cells, macrophages, and M2 polarized macrophages. Transcriptomic analysis predicted a trend toward more tendon fibroblasts 3 cells, corresponding to the *Col22a1*‐enriched junctional fibroblast population defined in the reference single‐cell dataset [39], in the tendons of ScxCre;Piezo1^f/f^ mice compared to WT mice. Based on this trend, we further investigated whether overall fibroblast abundance differed between genotypes using vimentin as a general fibroblast marker. We hypothesized that the higher cell density we discovered in the ScxCre;Piezo1^f/f^ mice would be positive for the fibroblast marker vimentin. We performed additional immunohistochemical analysis for vimentin combined with NucBlue. ScxCre;Piezo1^f/f^ tendons showed a higher mean percentage of vimentin^+^ cells within the defect region compared with WT tendons; however, this difference did not reach statistical significance (*p* = 0.31, Figure 4F–H).

These data suggest that the increased cellularity observed in ScxCre;Piezo1^f/f^ defect regions may partially reflect a greater abundance of vimentin^+^ fibroblast‐like stromal cells, although this difference did not reach statistical significance.

### 3.6 Loss of PIEZO1 Reduces ScxGFP Infiltration Into Defect Region After Injury

Our transcriptomic deconvolution analysis predicted a potential trend toward a smaller fraction of tendon fibroblasts 1 in defect tendons from ScxCre;Piezo1^f/f^ mice compared to WT mice. The tendon fibroblasts 1 subpopulation represents the *Spp1*‐enriched fibroblast population and is characterized by moderate to high *Col1a1* expression with *Scx* expression detected in a small portion of cells [39]. Therefore, we sought to further investigate the observed trend in smaller cell fraction by examining the number of Scx^+^ cells in the tendon defect by crossing our ScxCre;Piezo1^f/f^ mice with ScxGFP mice. Because previous literature identified day 10 as the peak period of Scx^+^ cell migration during tendon healing [28], we examined the ScxGFP mice at this timepoint.

The defected tendons from ScxGFP WT mice appeared to have more ScxGFP cells in the defect region than those from ScxCre;Piezo1^f/f^ mice (representative images Figure 5A,B, respectively). When examining the sham patellar tendons, we found that the ScxGFP signal in patellar tendons from both the WT and ScxCre;Piezo1^f/f^ mice appeared similar (representative images Figure 5C,D). Quantification of defected tendons revealed a significantly smaller Scx^+^ area in the defect region of the tendon from the ScxCre;Piezo1^f/f^ mice than in that from the WT mice, when Scx^+^ area was calculated as a percentage of NucBlue‐stained area (*p* = 0.006, Figure 5E). Consistent with the reduced ScxGFP‐positive area, *Scx* gene expression was significantly reduced in ScxCre;Piezo1^f/f^ defect tendons compared with WT (*p* < 0.001, Figure 5G). The change in defect width from the day of surgery to euthanasia on day 10 was significantly higher in the ScxCre;Piezo1^f/f^ mouse tendons (*p* = 0.04, Figure 5F), indicating the size of the wound in the tendon from the ScxCre;Piezo1^f/f^ mice had been reduced more than that from the WT. Interestingly, Piezo1 immunostaining showed a lower percentage of Piezo1^+^ cells in ScxCre;Piezo1^f/f^ sham tendons compared with the corresponding defect tendons, whereas the percentage of Piezo1^+^ cells in ScxCre;Piezo1^f/f^ defect tendons approached that observed in WT tendons (Figure S2). These differences did not reach statistical significance.

**FIGURE 5:**
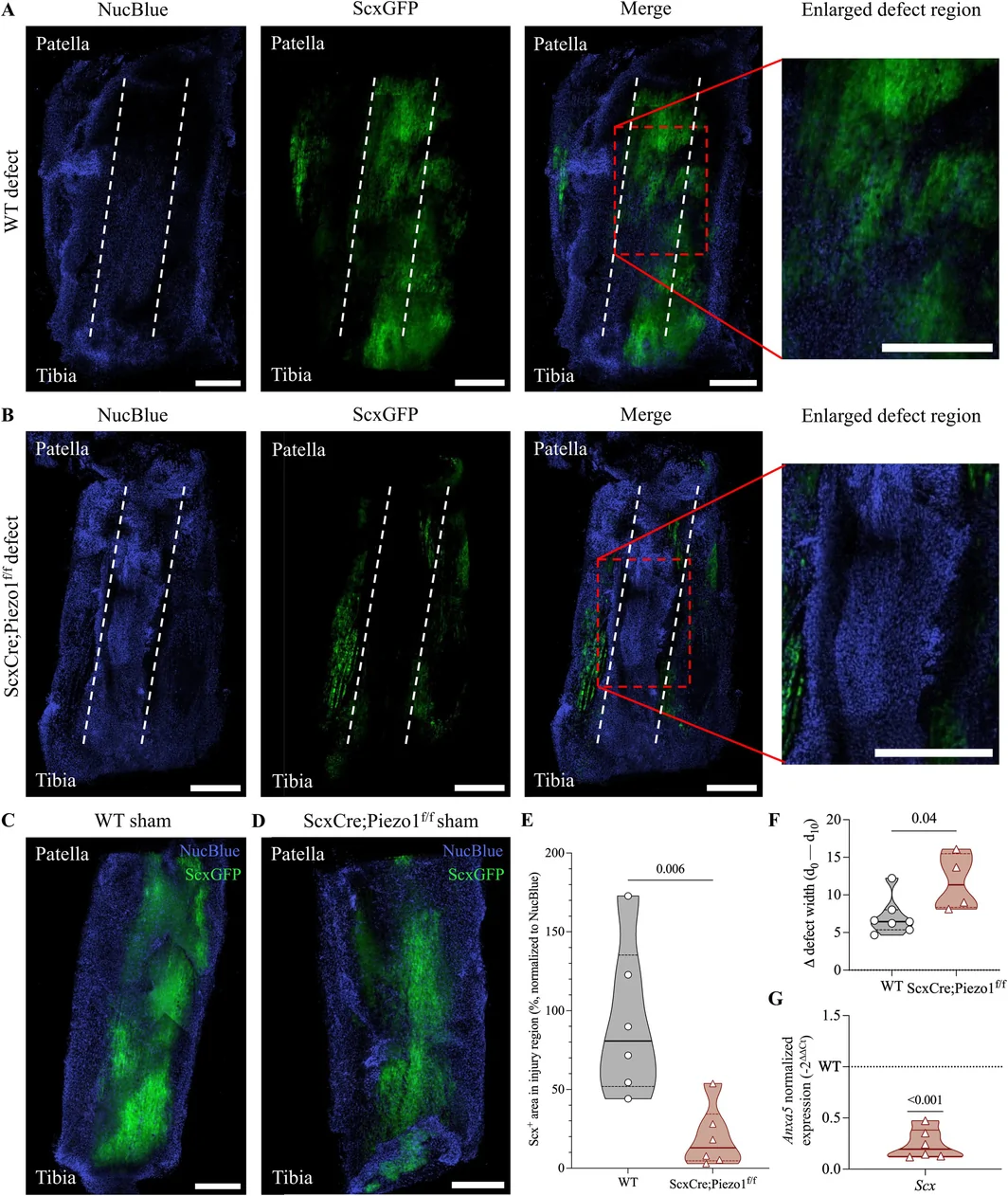
ScxCre;Piezo1^f/f^ mouse heals with reduced ScxGFP^+^ area in defect area than wildtype littermate control. (A) Representative z‐projection images of wildtype and (B) ScxCre;Piezo1^f/f^ mouse patellar tendons 10 days after full‐thickness central third defect surgery. Images from left to right are NucBlue staining, ScxGFP, merge, and enlargement of defect region. Dashed white line indicates the surgical defect. (C) Representative z‐projection images of wildtype and (D) ScxCre;Piezo1^f/f^ mouse contralateral sham patellar tendons. All scale bars represent 400 μm. (E) Quantification of Scx^+^ area in the defect region of the wounded patellar tendon normalized to that of the contralateral sham. Each point represents one mouse, *n* = 6 mice per group. Statistics performed with unpaired Student's *t*‐test and *p*‐value is shown. (F) Quantification of the change in defect width of the surgical injury calculated as the defect width on the day of surgery minus the defect width at euthanasia (day 10). Each point represents one mouse, *n* = 7 WT mice and 4 ScxCre;Piezo1^f/f^ mice. Statistics performed with unpaired Student's *t*‐test and *p*‐value is shown. (G) RT‐qPCR analysis of *Scx* in defect patellar tendons from ScxCre;Piezo1^f/f^ mice normalized to those from WT mice (day 14 post‐surgery). Each point represents one mouse, *n* = 6 mice. Statistics performed with one‐sample *t*‐test compared to WT value of 1, *p*‐value is shown.

Together, the vimentin and ScxGFP findings suggest that *Piezo1* depletion does not reduce overall stromal cell accumulation but alters the cellular composition of the reparative stromal response.

### 3.7 Scx^+^ Cell Migration In Vitro Is Reduced When *Piezo1* Is Silenced While Proliferation Remains Similar

After observing reduced ScxGFP signal within the defect region of ScxCre;Piezo1^f/f^ tendons, we next asked whether this reflected altered migration or proliferation of Scx^+^ cells. To explore this, an in vitro scratch assay was performed using *Piezo1* siRNA‐transfected patellar tendon cells (representative images in Figure 6A,B). *Piezo1*‐silenced cultures showed significantly fewer cells within the scratch area at each timepoint compared with scramble controls (Figure 6C). At 46 h, ScxGFP^+^ cells also represented a significantly smaller fraction of total cells within the scratch area in the *Piezo1*‐silenced group (Figure 6D). To determine whether reduced ScxGFP^+^ accumulation could instead reflect impaired proliferation, we isolated patellar tendon cells from ScxGFP WT and ScxGFP;ScxCre;Piezo1^f/f^ mice and cultured them over time. The number of ScxGFP^+^ cells was similar between groups at each timepoint (Figure 6E), suggesting that *Piezo1* depletion reduces Scx^+^ cell migration or wound‐area recruitment without detectably altering Scx^+^ cell proliferation. Consistent with the in vitro proliferation analysis, Ki67 immunostaining showed no significant difference in the percentage of proliferating cells within the defect region between genotypes (Figure S3).

**FIGURE 6:**
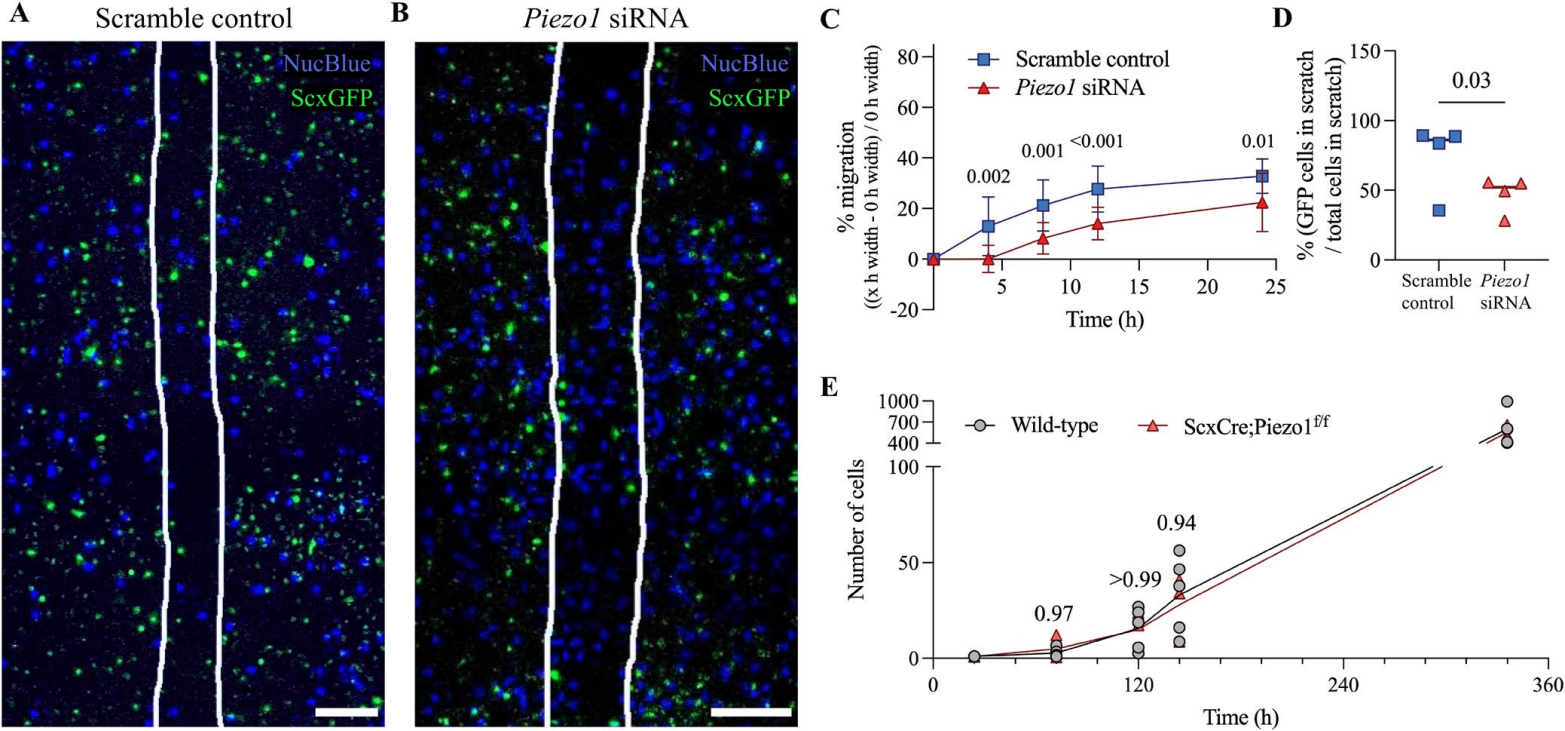
Silencing *Piezo1* affects the wound‐area repopulation/migration of Scx^+^ cells in vitro but not proliferation. Representative images of (A) scramble control cells and (B) *Piezo1* siRNA cells in a scratch assay after 46 h. White lines indicate original scratch boundaries. Green cells are ScxGFP^+^, and blue cells represent all cells. Scale bar represents 500 μm. (C) Migration of ScxGFP^+^ cells over time, indicated as a percentage of change in scratch width normalized to scratch width at time 0 h. Each point represents an average ± SD, *n* = 4 mice. (D) ScxGFP^+^ cells inside the original scratch borders at 46 h given as a percentage of total cells inside the scratch borders. Each dot represents an average of three replicates from one mouse, *n* = 4 mice. (E) Number of ScxGFP^+^ cells in a predefined area of a single well plated with mouse patellar tendon cells over time, normalized to time 0. Each point represents an average of two replicates per mouse, *n* = 5 WT mice and 3 ScxCre;Piezo1^f/f^ mice.

Taken together, these results indicate that *Piezo1* silencing reduces ScxGFP^+^ cell migration or wound‐area repopulation in vitro, without detectably affecting ScxGFP^+^ cell proliferation.

## 4 Discussion

Contrary to our original hypothesis, ScxCre‐driven *Piezo1* depletion did not impair patellar tendon healing. Instead, *Piezo1*‐depleted tendons showed improved mechanical recovery and more advanced collagen organization at early healing timepoints after full‐thickness central‐third defect injury. Bulk transcriptomic analysis showed that WT and ScxCre;Piezo1^f/f^ tendons activated overlapping core healing programs, although *Piezo1*‐depleted tendons exhibited broader transcriptional remodeling and selective enrichment of matrix organization signatures. Furthermore, reduced accumulation of Scx^+^ cells within the healing defect was accompanied by impaired wound‐area repopulation of *Piezo1*‐depleted Scx^+^ cells in vitro, suggesting that altered Scx^+^ cell migration may contribute to the observed healing phenotype.

Tensile testing of the patellar tendons 2 and 4 weeks post‐surgery revealed significantly higher sham‐normalized elastic modulus in the ScxCre;Piezo1^f/f^ defect tendons compared with WT defect tendons. The genotype difference was larger at 2 weeks than at 4 weeks, suggesting that WT tendons recovered material‐level mechanical properties more slowly than ScxCre;Piezo1^f/f^ tendons. Consistent with this interpretation, TEM analysis at 4 weeks showed features of more advanced structural remodeling in ScxCre;Piezo1^f/f^ tendons, including significantly higher collagen fibril density within the defect region and a nonsignificant shift toward a greater proportion of larger‐diameter fibrils. Picrosirius red staining further supported this structural phenotype, with ScxCre;Piezo1^f/f^ tendons showing a significantly larger area of red birefringence, consistent with thicker and/or more mature collagen fibers, whereas WT tendons showed a larger area of yellow birefringence, consistent with thinner and/or less mature collagen fibers. In parallel, whole transcriptomic analysis identified a greater number of significantly upregulated collagen genes in ScxCre;Piezo1^f/f^ defect tendons compared with sham tendons than in WT defect tendons compared with sham tendons. The transcriptomic data also suggested altered expression of extracellular matrix remodeling enzymes, including both collagen‐associated genes and matrix proteases. Together, these mechanical, ultrastructural, histological, and transcriptomic findings support the conclusion that *Piezo1* depletion accelerates matrix remodeling and structural recovery after patellar tendon injury.

Whole transcriptomic analysis revealed substantial overlap in core healing‐associated pathways between WT and ScxCre;Piezo1^f/f^ defect tendons, including extracellular matrix, basement membrane, Golgi, and endoplasmic reticulum‐associated terms. However, genotype‐specific enrichment patterns suggested differences in the repair state. WT defect tendons were selectively enriched for pathways associated with early reparative signaling, including glycosaminoglycan biosynthesis, p53‐related responses, cAMP metabolism, and PI3K/AKT signaling. In contrast, ScxCre;Piezo1^f/f^ defect tendons showed enrichment of protein trafficking and matrix organization pathways, including COPI‐related terms and collagen fibril organization. These signatures support the interpretation that *Piezo1*‐depleted tendons had progressed toward a more matrix‐organizing repair state, although this remains an inference from bulk tissue transcriptomics. Interestingly, pathway enrichment analysis also identified significant enrichment of biological processes related to cartilage development, regulation of chondrocyte differentiation, and collagen fibril organization in ScxCre;Piezo1^f/f^ healing tendons. Although several extracellular matrix genes contributing to these pathways were also present in WT healing tendons (*Col2a1, Col11a1/2, Col9a1/2/3, Fgfr3*, etc.), enrichment reached statistical significance only in the ScxCre;Piezo1^f/f^ dataset. These findings suggest PIEZO1 depletion alters extracellular matrix remodeling programs during tendon healing. However, pathway enrichment alone does not establish chondrogenic differentiation or cartilage formation within the healing tendon. Rather, these pathways may reflect activation of transcriptional programs shared between tendon remodeling, fibrocartilage formation, and skeletal development. Additional studies incorporating cartilage‐specific histology, immunostaining, or single‐cell transcriptomics will be required to determine the biological significance of these findings.

Cellular deconvolution of the bulk transcriptomic data predicted no significant differences in cell population fractions between WT and ScxCre;Piezo1^f/f^ defect tendons. The predicted cell populations correspond to transcriptionally distinct clusters identified in the published murine Achilles tendon single‐cell RNA sequencing dataset used as the reference for CIBERSORTx analysis. In that dataset, the tendon fibroblast subpopulations were characterized by moderate‐to‐high levels of *Col1a1* but differed in the expression of additional extracellular matrix‐associated genes, including enrichment of *Spp1* in tendon fibroblast 1, *Dpt* in tendon fibroblast 2, and *Col22a1* in the junctional fibroblast population corresponding to tendon fibroblast 3 in our analysis [39]. Endothelial and immune cell populations were defined by expression of canonical markers including *Pecam1* (CD31) and *Ptprc* (CD45), respectively, with the immune population also enriched for *Mrc1* (CD206) [39]. Consistent with the deconvolution analysis, whole mount immunohistochemistry showed no genotype‐dependent differences in CD31^+^ or CD206^+^ area within the defect region. Thus, the accelerated structural and functional recovery observed in ScxCre;Piezo1^f/f^ tendons was not associated with detectable differences in endothelial or CD206^+^ macrophage accumulation.

Transcriptomic deconvolution suggested genotype‐dependent trends in tendon fibroblast subpopulations, prompting further analysis of stromal cell composition within the defect region. Immunohistochemistry for vimentin, used here as a broad marker of mesenchymal/stromal cells, showed a higher mean percentage of vimentin^+^ cells in the defect region of ScxCre;Piezo1^f/f^ tendons compared with WT tendons, although this difference did not reach statistical significance. Sham tendons showed similar vimentin staining between genotypes. These findings suggest that *Piezo1* depletion does not reduce overall stromal cell accumulation within the healing defect. However, cellular deconvolution also suggested a trend toward a lower fraction of tendon fibroblasts 1 cells in ScxCre;Piezo1^f/f^ defect tendons, a subpopulation reported to include a subset of *Scx*‐expressing cells [39]. In addition, PI3K/AKT signaling, which was selectively enriched in WT defect tendons, has been implicated in Scx‐lineage cell migration [45], whereas TNF‐α signaling, selectively enriched in ScxCre;Piezo1^f/f^ defected tendons, has been reported to suppress *Scx* and other tenogenic markers [46]. We therefore crossed ScxCre;Piezo1^f/f^ mice with ScxGFP reporter mice to directly evaluate *Scx*‐associated signal within the healing defect. Whole mount imaging at day 10 revealed a smaller ScxGFP^+^ area in the central defect region of ScxCre;Piezo1^f/f^ tendons compared with WT tendons. This reduced ScxGFP signal indicated reduced accumulation and/or maintenance of *Scx*‐expressing cells within the defect region. Interestingly, despite the reduced ScxGFP^+^ area, Piezo1^+^ cells remained abundant in ScxCre;Piezo1^f/f^ defects, approaching levels observed in WT tendons (Figure S2). Although this observation did not reach statistical significance, it may suggest a shift toward *Piezo1*‐expressing cells outside the ScxCre‐targeted population within the healing defect. In vitro assays further suggested that the reduction in ScxGFP^+^ cells was more consistent with impaired migration or wound‐area repopulation than with reduced proliferation. Supporting this interpretation, Ki67 immunostaining showed similar percentages of proliferating cells across groups (Figure S3). These findings are consistent with recent work showing that depletion of Scx‐lineage cells before digital flexor tendon injury improves functional healing outcomes [47], and support a model in which reduced Scx‐lineage cell accumulation may contribute to improved tendon repair in ScxCre;Piezo1^f/f^ mice. Future studies should determine whether the reduced wound‐area repopulation observed following *Piezo1* depletion is associated with alterations in cytoskeletal organization, focal adhesion dynamics, or other biomechanical properties that regulate directed cell migration.

Previous studies indicate that *Scx* expression and Scx‐lineage cell function are dynamically regulated during tendon healing [47, 48]. The functional contribution of Scx‐lineage cells appears to be temporally dependent: depletion of Scx‐lineage cells before injury improved subsequent functional healing [47], whereas depletion during the proliferative phase has been shown to impair restoration of tendon structure and mechanics [49]. These findings support the possibility that the reduced accumulation of Scx^+^ cells observed in ScxCre;Piezo1^f/f^ tendons may have distinct functions at different stages of repair. Importantly, *Scx* regulation during tendon healing is closely linked to the mechanical environment and TGF‐β signaling. Mechanical loading can regulate the release of active TGF‐β from the tendon ECM, and following tendon injury, TGF‐β signaling has been shown to induce *Scx* expression in recruited progenitor cells [50, 51]. Thus, changes in the mechanical environment during healing may influence both *Scx* expression and cell recruitment, in part through TGF‐β‐dependent mechanisms.

The reduced *Scx* signal in ScxCre;Piezo1^f/f^ tendons may also reflect altered signaling within the healing microenvironment rather than solely impaired cell recruitment. Wnt/β‐catenin signaling has been shown to become activated following tendon injury to suppress *Scx* and other tenogenic genes in tendon‐derived cells [52], while TNF‐α can similarly reduce *SCX* expression in tenocytes [53]. Because TNF‐α signaling was enriched in ScxCre;Piezo1^f/f^ defect tendons and Wnt/β‐catenin signaling was also among the enriched pathways identified in our transcriptomic analysis, altered inflammatory and Wnt signaling could contribute to the reduced *Scx* expression observed following *Piezo1* depletion. However, the present study cannot distinguish altered *Scx* expression within resident cells from altered recruitment or maintenance of Scx^+^ cells. The reduced accumulation of Scx^+^ cells and accelerated matrix remodeling observed following *Piezo1* depletion may therefore reflect integration of multiple mechanosensitive pathways rather than an isolated effect of PIEZO1 signaling. Resolving these mechanisms will require spatial and temporally controlled analyses.

One potential mechanism underlying the altered migration of Scx^+^ cells is PIEZO1‐dependent durotaxis. PIEZO1 has been implicated in mechanically directed cell migration, and similar context‐dependent effects have been reported in skin wound healing, where *Piezo1* conditional KO accelerated wound closure whereas PIEZO1 activation slowed keratinocyte migration [54]. Although tendon healing involves distinct cell populations and extracellular matrix mechanics, these findings support the broader concept that PIEZO1‐mediated mechanosensing can regulate reparative cell migration in a tissue‐ and context‐dependent manner. Whether the reduced accumulation of Scx^+^ cells observed here specifically reflects altered durotaxis remains to be determined.

This study had limitations. We used only female mice in this study because, in preliminary experiments, we found that male mice exhibited improper healing about 80% of the time. Therefore, to reduce the number of mice required for the study, we decided to focus only on female mice. While we expect the same trends regarding *Piezo1* and *Scx* to hold true for both sexes, we would need to confirm this in future studies. Additionally, the patellar tendon injury model involves a precise cut in the tendon, whereas most tendon injuries occur due to tearing the tissue, which limits the translatability of the model. One exception to this is ACL reconstruction surgery, where the central third of the patellar tendon is surgically removed with bony pieces to be used as an autograft [20, 21, 22, 23]. However, our animal model is an accepted model of tendon injury because it recapitulates the partial unloading that occurs during human tendon healing without the need for casting. Although the contralateral limb underwent sham surgery to minimize compensatory loading during recovery, gait, limb loading, and overall activity were not quantitatively assessed. Consequently, transient postoperative alterations in mechanical loading of either limb cannot be excluded and may have influenced the local mechanical environment experienced by the tendon. In our histological analysis, it was difficult to know exactly where in the tendon we had sectioned due to the orientation of the sectioning. While we did our best to approximate the defected region of the tendon, the method of sectioning did not allow us to be certain. Additionally, PSR birefringence may be influenced by collagen crimp and by fiber orientation relative to the sectioning and polarization planes. Although all hindlimbs were fixed in a standardized 90° configuration and processed and imaged identically, subtle differences in tendon orientation within the sections cannot be completely excluded as a source of variability. Histological analysis was also quantified across the entire healing defect, rather than within predefined anatomical subregions. Although this approach allowed standardized comparison between genotypes, it may have masked regional heterogeneity. Future studies incorporating region‐specific analyses across the defect and adjacent tendon tissue may provide additional insight into the spatial regulation of tendon healing. Another limitation is that different experimental endpoints were assessed at different stages of healing rather than across a common longitudinal timeline. The selected timepoints were chosen to capture distinct biological processes based on prior literature. Mechanical testing was performed at 2 and 4 weeks to assess functional recovery during tissue remodeling, whereas ScxGFP analysis was performed at 10 days to capture the reported peak migration of Scx‐lineage cells during tendon healing. Consequently, direct temporal comparisons between all experimental outcomes are not possible. Furthermore, the RNA yield from a patellar tendon is low, necessitating the pooling of tendons for RNA sequencing. Cellular deconvolution was performed using a publicly available single‐cell RNA sequencing dataset from male murine Achilles tendon as the reference dataset [39]. Although we confirmed these findings with immunohistochemistry, future studies using patellar tendon‐specific single‐cell datasets would further strengthen these analyses. Another limitation was that the entire defect tendon, including intact struts and defected region, was taken and processed for whole transcriptomic analysis. This was done to ensure a proper RNA yield for sequencing; however, the inclusion of the intact struts in the analysis likely masked differences unique to the central‐third healing region. Spatial transcriptomics, microdissection of the defect region, or single‐cell RNA‐seq would be needed to resolve cell‐type‐specific and defect‐localized transcriptional effects of *Piezo1* depletion. Additionally, as with all animal studies, considerable variation was observed in the data. We were able to use minimal animals and see significant differences, which gives credence to our results. However, it is still important to acknowledge the large variation. A final limitation is that ScxCre‐driven *Piezo1* conditional KO does not distinguish between developmental effects in Scx‐lineage cells and acute PIEZO1 function during repair. Therefore, the improved healing phenotype may reflect altered baseline properties of Scx‐lineage cells, altered recruitment during injury, or both. Inducible, temporally controlled *Piezo1* deletion would be needed to isolate the role of PIEZO1 during the healing phase itself.

Taken together, our findings reveal a context‐dependent role for PIEZO1 in tendon mechanobiology. We previously identified PIEZO1 as a shear stress sensor that supports extracellular matrix remodeling and mechanical adaptation during tendon homeostasis [17]. In contrast, the present data suggest that PIEZO1 activity in Scx‐lineage cells can delay structural and functional recovery after tendon injury. One possible explanation is that PIEZO1 promotes migration or wound‐site accumulation of Scx^+^ reparative cells at the injury site. If this population preferentially contributes to fibrotic, rather than regenerative repair, reduced recruitment in ScxCre;Piezo1^f/f^ mice could contribute to the improved mechanical recovery observed in the present study. However, the healing environment differs fundamentally from tendon homeostasis and incorporates inflammatory, mechanical, and lineage‐specification signals that may modify the consequences of PIEZO1 activation. In particular, the enrichment of TNF‐α signaling observed in ScxCre;Piezo1^f/f^ defect tendons, together with the known interactions between inflammatory signaling and tenogenic cell phenotype, suggests that PIEZO1‐dependent mechanosensing may interact with the inflammatory environment during repair. Collectively, our findings support the hypothesis that PIEZO1 functions as a context‐dependent regulator of multiple interconnected aspects of tendon repair. In addition to reduced accumulation of Scx^+^ cells, ScxCre;Piezo1^f/f^ tendons exhibited improved collagen organization and mechanical recovery together with enrichment of cartilage development‐ and chondrocyte differentiation‐associated transcriptional programs. These observations may represent coordinated downstream consequences of altered mechanosensing following *Piezo1* depletion rather than independent biological events. However, the present study cannot determine whether these findings are causally related or represent parallel effects of altered PIEZO1 signaling. Future studies will be required to establish temporal and mechanistic relationships among cell recruitment, extracellular matrix remodeling, and lineage‐associated transcriptional programs during tendon healing, and to determine how PIEZO1 coordinates these processes. Thus, PIEZO1 appears to have distinct roles in homeostatic tendon adaptation and reparative remodeling after injury.

These findings raise the possibility that transient modulation of PIEZO1 activity could influence tendon repair. However, therapeutic translation will require substantial additional work, including temporally‐controlled pharmacological inhibition studies to determine whether PIEZO1 antagonism can reproduce the genetic phenotype without compromising tendon homeostasis, matrix quality, or long‐term mechanical function. This work therefore identifies PIEZO1‐dependent Scx^+^ cell recruitment as a candidate mechanism regulating tendon healing outcomes and opens a new avenue for investigating mechanosensitive control of tendon repair.

## Author Contributions

N.A.C. designed the studies, performed experiments, analyzed the data, and wrote the manuscript. M.W. performed all transcriptomic analyses. M.B.‐Y.G. helped with in vitro experiments and assisted in some surgeries. M.B. assisted with in vitro experiments. J.G.S. designed the study, analyzed the data, and edited the manuscript. All authors have read and approve the manuscript.

## Funding

This work was supported by Schweizerischer Nationalfonds zur Förderung der Wissenschaftlichen Forschung (SNF), 185095, 207489, and 10000132.

## Conflicts of Interest

The authors declare no conflicts of interest.

## Supporting information


**Figure S1:** Generation of a ScxCre;Piezo1^f/f^ mouse. (A) Schematic representation of genetic modification of the mouse model with *Piezo1* being surrounded by flox sequences, which were used to excise the gene in Scx^+^ cells using a ScxCre driver. Representative images of patellar tendon sections from (B) wild‐type and (C) ScxCre;Piezo1^f/f^ mice stained with Piezo1 antibody (red) and NucBlue (blue). Patellar tendon is circled in white and scale bars represent 400 μm. Zoom magnification of selected areas in yellow, scale bars represent 200 μm. (D) Quantification of Piezo1^+^ stained area normalized to total nuclear area in the patellar tendon. Statistical analysis was performed with a *t*‐test, and each point represents an average of 2 sections from one mouse, *n* = 3 mice. (E) Quantification of *Piezo1* gene expression in patellar tendon (PT) and tail tendon fascicles (TTF) of ScxCre;Piezo1^f/f^ mice normalized to wild‐type levels (1.0). Statistical analysis was performed with a one‐sided *t*‐test compared to the WT value of 1.0, and each point represents one mouse, *n* = 3.
**Figure S2:** Piezo1 immunohistochemistry following full‐thickness central‐third patellar tendon defect surgery. Representative images of (A) ScxCre;Piezo1^f/f^ defect, (B) ScxCre;Piezo1^f/f^ sham, (C) WT defect, and (D) WT sham tendons. Scale bars represent 20 μm. Nuclei are represented in blue and Piezo1^+^ nuclei are surrounded by green. Red triangles indicate examples of Piezo1^+^ cells. (E) Quantification of Piezo1^+^ cells as a percentage of nuclei. Bars are represented as average ± standard deviation. Each point represents an average of four technical replicates for one mouse (*n* = 3).
**Figure S3:** Ki67 immunohistochemistry following full‐thickness central‐third patellar tendon defect surgery. Representative images of (A) ScxCre;Piezo1^f/f^ defect, (B) ScxCre;Piezo1^f/f^ sham, (C) WT defect, and (D) WT sham tendons. Scale bars represent 20 μm. Nuclei are represented in blue and Ki67^+^ nuclei are surrounded by red. Yellow triangles indicate examples of Ki67^+^ cells. (E) Quantification of Ki67^+^ cells as a percentage of nuclei. Bars are represented as average ± standard deviation. Each point represents an average of four technical replicates for one mouse (*n* = 3).

## Data Availability

Most data that support the findings of this study are available in the Results and Supporting Information of this article. The RNA‐sequencing data generated in this study have been deposited in the NCBI Gene Expression Omnibus (GEO) under the accession number GSE346704. Any data not included are available on request from the corresponding author.
