# Supplementary material for: PIEZO1 Is a Contextual Mechanosensor and Its Depletion in Scx‐Lineage Cells Accelerates Structural and Functional Recovery
Source: FASEB J. 2026 Oct 7;40(19):e72360. doi: 10.1096/fj.202602931R (PMC13655483; doi:10.1096/fj.202602931R)
Supplement: Supplementary file 1 [file FSB2-40-e72360-s001.docx]

**PIEZO1 is a contextual mechanosensor and its depletion during healing accelerates structural and functional recovery**

Nicole A. Chittim^1,2^, Maja Wolleb^1,2^, Maya Ben-Yehuda Greenwald^1,2^, Maja Bollhalder^1,2^, Jess G. Snedeker^1,2^

^1^ Department of Orthopedics, Balgrist University Hospital Zurich, University of Zurich, Switzerland

^2^ Institute for Biomechanics, ETH Zurich, Zurich, Switzerland

**Supplementary Information**

**Whole mount immunostaining buffers**

| Buffer | Content |
| --- | --- |
| Antigen retrieval buffer | 25% urea, 15% glycerol, and 7% Triton-X100 in deionized water |
| Blocking buffer | 10% donkey serum, 10% DMSO, and 0.5% Triton-X100 in PBS |
| Primary Ab solution | 2% donkey serum, 10% DMSO, and 0.5% Triton-X100 in PBS with primary Ab’s |
| Wash buffer | 2% donkey serum and 0.5% Triton-X100 in PBS |
| Secondary Ab solution | 2% donkey serum, 10% DMSO, and 0.5% Triton-X100 in PBS |

**Supplementary Figures**

**Supplementary Fig. 1. Generation of a ScxCre;Piezo1^f/f^ mouse.** (A) Schematic representation of genetic modification of the mouse model with *Piezo1* being surrounded by flox sequences, which were used to excise the gene in Scx^+^ cells using a ScxCre driver. Representative images of patellar tendon sections from (B) wild-type and (C) ScxCre;Piezo1^f/f^ mice stained with Piezo1 antibody (red) and NucBlue (blue). Patellar tendon is circled in white and scale bars represent 400 μm. Zoom magnification of selected areas in yellow, scale bars represent 200 μm. (D) Quantification of Piezo1^+^ stained area normalized to total nuclear area in the patellar tendon. Statistical analysis was performed with a t-test, and each point represents an average of 2 sections from one mouse, n = 3 mice. (E) Quantification of *Piezo1* gene expression in patellar tendon (PT) and tail tendon fascicles (TTF) of ScxCre;Piezo1^f/f^ mice normalized to wild-type levels (1.0). Statistical analysis was performed with a one-sided t-test compared to the WT value of 1.0, and each point represents one mouse, n = 3.

**Supplementary Fig. 2. Piezo1 immunohistochemistry following full-thickness central-third patellar tendon defect surgery.** Representative images of (A) ScxCre;Piezo1^f/f^ defect, (B) ScxCre;Piezo1^f/f^ sham, (C) WT defect, and (D) WT sham tendons. Scale bars represent 20 μm. Nuclei are represented in blue and Piezo1^+^ nuclei are surrounded by green. Red triangles indicate examples of Piezo1^+^ cells. (E) Quantification of Piezo1^+^ cells as a percentage of nuclei. Bars are represented as average +/- standard deviation. Each point represents an average of four technical replicates for one mouse (n = 3).

**Supplementary Fig. 3. Ki67 immunohistochemistry following full-thickness central-third patellar tendon defect surgery.** Representative images of (A) ScxCre;Piezo1^f/f^ defect, (B) ScxCre;Piezo1^f/f^ sham, (C) WT defect, and (D) WT sham tendons. Scale bars represent 20 μm. Nuclei are represented in blue and Ki67^+^ nuclei are surrounded by red. Yellow triangles indicate examples of Ki67^+^ cells. (E) Quantification of Ki67^+^ cells as a percentage of nuclei. Bars are represented as average +/- standard deviation. Each point represents an average of four technical replicates for one mouse (n = 3).

**ARRIVE Guidelines Information**

| **General information for all experiments involving mouse surgery** | | |
| --- | --- | --- |
| Methods: study design | Randomization / blinded | Randomized |
|  | Experimental unit | Group-housed |
| Methods: experimental procedures | Experiment description | Full-thickness, central-third defect surgery on left leg with right leg undergoing sham surgery. Returned to home cage after recovery from anesthesia. Euthanasia of mice at pre-defined timepoints based on specific esxperiment and tendons isolated. |
|  | Time of day for experiment | Surgery beginning in the morning at approximately 7:30. |
|  | Where experiment was performed | Surgery in animal laboratory, returned to home cage for recovery and duration of experiment. |
|  | Rationale for anesthetic | Buprenorphine for anelgesia because it was not expected to interfere with clinical results (consultation with Prof. Dr. med Vet. PhD Regula Bettschart-Wolfensberger, head of section anesthesiology, Animal Hospital, University of Zurich, Switzerland). Inhalation isoflurane for surgical anesthesia because it requires minimal animal handling, has rapid induction, is easy to administer, and has a quick recovery time with low risk of complication. |
| Methods: experimental animals | Animals used (species, strain, sex, age) | C57BL/6, females only, aged 16-22 weeks old (adult mice).  Genotypes: ScxCre;Piezo1^f/f^, Piezo1^f/f^ (WT), ScxGFP;ScxCre;Piezo1^f/f^, ScxGFP;Piezo1^f/f^ (ScxGFP WT) |
|  | Source of animals | Internal breeding, generation F12+ |
| Methods: housing & husbandry | Housing (SPF, type of cage, bedding, number of cage mates) | NexGen IVC 500 mouse cages. Premium scientific bedding (SAFE, Augy, France). 2-4 cage mates |
|  | Husbandry (breeding, light/dark cycle, temperature, type of food, access to food and water, environmental enrichment) | 12 hour light/dark cycle. Room temperature 21 degrees C. Standard rodent chow (Kliba Nafag, Kaiseraugst, Switzerland). Access to food and water: ad libitum. Crinkle paper (SAFE crinklets natural, SAFE, Augy, France), tissue, and red mouse house in cage for enrichment. |
|  | Welfare assessments | Days 1-4 post-surgery: mice checked daily for weight, surgical site, gait, and behavior. After 4 days: mice checked weekly for the same parameters. In case of pain symptoms, mice were treated with buprenorphine. |
| Methods: allocating animals | Animal allocation to groups | Animals within a genotype were randomly allocated. |
|  | Order of treatment for animals | Surgery was performed blinded to genotype. |
| Results: adverse events | Adverse events | None |

| **Specific information based on experimental outcome** | | |
| --- | --- | --- |
| Experimental outcome: Mechanical properties | | |
| Methods: study design | Number of animals | 28 total (n = 7 mice / genotype / timepoint) |
|  | Timepoint for euthanasia | 2 weeks or 4 weeks post-surgery |
| Methods: sample size | Number of independent replicates | Surgery performed 2 independent times per timepoint |
|  | Rationale for sample size | Calculations performed based on a possible scenario that we were looking for at least a 20% difference in group mean with a stnadard deviation of 10%. Sample size calculations allowed for 80% power analysis. |
| Methods: statistical methods | Statistical methods | Students unpaired t-test (normality confirmed with Q-Q plot) |
|  | Unit analysis for statistics | Single hindleg defect normalized to corresponding sham from one animal. |
| Experimental outcome: Transmission electron microscope | | |
| Methods: study design | Number of animals | 6 mice total (n = 3 mice / genotype) |
|  | Timepoint for euthanasia | 4 weeks post-surgery |
| Methods: sample size | Number of independent replicates | Surgery performed once |
|  | Rationale for sample size | Calculations performed based on a possible scenario that we were looking for at least a 20% difference in group mean with a stnadard deviation of 10%. Sample size calculations allowed for 80% power analysis. |
| Methods: statistical methods | Statistical methods | Students unpaired t-test (normality confirmed with Q-Q plot) |
|  | Unit analysis for statistics | Portion of tendon (intact strut or defect) from one animal. |
| Experimental outcome: Histology (H&E, PSR, Vim, Piezo1) | | |
| Methods: study design | Number of animals | 3 mice / genotype for each stain |
|  | Timepoint for euthanasia | 2 weeks post-surgery |
| Methods: sample size | Number of independent replicates | Surgery performed once |
|  | Rationale for sample size | Calculations performed based on a possible scenario that we were looking for at least a 20% difference in group mean with a stnadard deviation of 10%. Sample size calculations allowed for 80% power analysis. |
| Methods: statistical methods | Statistical methods | Students unpaired t-test when normality confirmed with Q-Q plot, otherwise nonparametric test |
|  | Unit analysis for statistics | Average of 3-4 sections from one hindlimb from one animal. |
| Experimental outcome: whole transcriptomic analysis | | |
| Methods: study design | Number of animals | 24 total (n = 6 mice / genotype, pooled 2 tendons for 1 sample) |
|  | Timepoint for euthanasia | 2 weeks post-surgery |
| Methods: sample size | Number of independent replicates | Surgery performed 2 independent times |
|  | Rationale for sample size | Calculations performed based on a possible scenario that we were looking for at least a 20% difference in group mean with a stnadard deviation of 10%. Sample size calculations allowed for 80% power analysis. |
| Methods: statistical methods | Statistical methods | SUSHI platform |
|  | Unit analysis for statistics | Analysis is defect leg vs sham for both genotypes. Two tendons of each type were pooled for processing. |
| Experimental outcome: Whole mount analysis (IHC & ScxGFP) | | |
| Methods: study design | Number of animals | 24 mice total (n = 6 mice / genotype / analysis) |
|  | Timepoint for euthanasia | 2 weeks post-surgery for IHC, 10 days post-surgery for ScxGFP |
| Methods: sample size | Number of independent replicates | Surgery performed 2 independent times for each analysis |
|  | Rationale for sample size | Calculations performed based on a possible scenario that we were looking for at least a 20% difference in group mean with a stnadard deviation of 10%. Sample size calculations allowed for 80% power analysis. |
| Methods: statistical methods | Statistical methods | Students unpaired t-test when normality confirmed with Q-Q plot, otherwise nonparametric test |
|  | Unit analysis for statistics | IHC: Single hindleg defect normalized to corresponding sham from one animal.  ScxGFP: Single defect hindlimb |
